# Silicon Field Effect Transistor as the Nonlinear Detector for Terahertz Autocorellators

**DOI:** 10.3390/s18113735

**Published:** 2018-11-02

**Authors:** Kęstutis Ikamas, Ignas Nevinskas, Arūnas Krotkus, Alvydas Lisauskas

**Affiliations:** 1 Institute of Applied Electrodynamics and Telecommunications, Vilnius University, Sauletekio av. 3, LT-10257 Vilnius, Lithuania; 2 The General Jonas Žemaitis Military Academy of Lithuania, Šilo str. 5A, LT-10322 Vilnius, Lithuania; 3 Center For Physical Sciences And Technology, Sauletekio av. 3, LT-10257 Vilnius, Lithuania; ignas.nevinskas@ftmc.lt (I.N.); arunas.krotkus@ftmc.lt (A.K.)

**Keywords:** detector, FET, interference, nonlinear effect, THz, transistor

## Abstract

We demonstrate that the rectifying field effect transistor, biased to the subthreshold regime, in a large signal regime exhibits a super-linear response to the incident terahertz (THz) power. This phenomenon can be exploited in a variety of experiments which exploit a nonlinear response, such as nonlinear autocorrelation measurements, for direct assessment of intrinsic response time using a pump-probe configuration or for indirect calibration of the oscillating voltage amplitude, which is delivered to the device. For these purposes, we employ a broadband bow-tie antenna coupled Si CMOS field-effect-transistor-based THz detector (TeraFET) in a nonlinear autocorrelation experiment performed with picoseconds-scale pulsed THz radiation. We have found that, in a wide range of gate bias (above the threshold voltage $V_{\mathrm{th}}=445$ mV), the detected signal follows linearly to the emitted THz power. For gate bias below the threshold voltage (at 350 mV and below), the detected signal increases in a super-linear manner. A combination of these response regimes allows for performing nonlinear autocorrelation measurements with a single device and avoiding cryogenic cooling.

## 1. Introduction

The development or maintenance of pulsed electromagnetic radiation sources requires some special techniques to supervise their temporal characteristics. Sources that operate in the optical and mid-infrared spectral domains are often monitored by the interferometric intensity autocorrelation technique [1,2]. For the intensity autocorrelation, one often employs quadratic (to the incident power) detectors, two-photon absorption or sum-frequency generation. In the THz frequency domain, one option is quantum-engineered devices based on intersubband transitions; however, such detectors require cryogenic cooling [3]. In addition, antenna-coupled field-effect transistors (FET) and the Schottky diodes have recently emerged as useful devices for room-temperature THz autocorrelators [4,5]. We have recently demonstrated that FETs biased in the sub-threshold regime in a certain range of excitation amplitudes exhibit a super-linear response [6].

Pulsed THz sources that are based on either photomixing or optical rectification driven by a femtosecond laser usually exhibit low average power levels (up to several of μW), originating from the low conversion efficiency of the emitter. The so-called Large-Area Photoconductive antennas produce up to 1 mW of radiation power at best, but such devices are driven by a low repetition rate and high pulse power femtosecond lasers [7,8]. Further improvements in photoconductive antennas take advantage of the surface plasmon polariton phenomena [9] or the usage of the hybrid graphene molybdenum disulphide structure [10].

Despite low average power levels, traditional THz photoconductive antennas can reach watt levels at the peak power ([11], p. 66), [12,13]. Therefore, these sources can serve as a useful tool to investigate the response of fast detectors as a function of power of THz radiation. Although detecting THz pulses with photoconductive antennas provide several advantages over conventional power detectors such as preserved phase information and relatively high signal-to-noise ratios [14], these detectors are not suited to trace radiation power. Moreover, even the ultrashort (below 1 ps) carrier trapping time of the epitaxial layers [15] distort the original THz pulse reducing system bandwidth overall [16].

Here, we employ the autocorellation technique and investigate the response of the FET detector to THz excitation with pulse duration being close to a single oscillation cycle.

## 2. Si CMOS FET Detector

**Figure 1:**
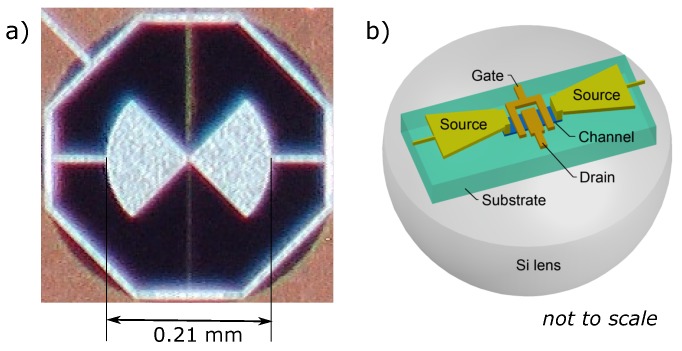
(**a**) the micrograph image of the bow-tie detector on a CMOS crystal is used in this paper; (**b**) a simplified schematic view of the detector (not to scale).

In this study, we employ a broadband bow-tie antenna-coupled THz detector based on a CMOS (complementary metal-oxide-semiconductor) FET (TeraFET). The device was fabricated using a commercial 90 nm silicon CMOS process of the Taiwan Semiconductor Manufacturing Company (TSMC) provided by Europractice service. A double-transistor layout was chosen as in earlier reported designs with CMOS FETs [17,18,19]. We selected a so-called ultra-low threshold voltage ($V_{\mathrm{th}}$ = 445 mV) NMOS (n-channel metal-oxide-semiconductor field-effect transistor) transistors with a channel length of 100 nm and a width of 500 nm each. Figure 1 presents the micrograph of a broadband bow-tie detector and its simplified schematic view. The THz radiation is coupled from the bottom of the structure through a hyper-hemispherical silicon lens (12 mm diameter and 7.2 mm height with additional 440-μm-thick substrate) and a reduced 280 μm-thick p-doped Si substrate of the chip, and is received by a bow-tie antenna, which is integrated into the top metal/insulator stack of the CMOS process. The antenna has an opening angle of 90${}^{\circ }$, the length of each leaf is 105 μm. We designed this antenna to have nearly flat impedance for frequencies beyond 2 THz. The THz signal is guided downwards vertically to NMOS transistors using vias as feeds. The rectified voltage which builds-up between a source and a drain is taken for a readout through the conductor wire, located in the virtual ground plane at the axis of symmetry of the antenna and the ends of electrically connected antenna leaves. We do not bias the transistor channel; therefore, the amplitude of electrical fluctuations $V_{N}$ is limited by the Johnson noise [20].

The TeraFET design was optimized using an in-house developed physics based circuit model [18,21], which allows for identifying the role of intrinsic rectification and circuit elements and optimizing the performance of the device. This model based on the nonlinear plasma wave mixing mechanism of rectification in the FETs channel was formulated for the first time by Dyakonov and Shur [22] and has been exploited by many research groups [18,23,24,25]. For modeling the responsivity and the optical noise equivalent power (NEP), we used these device parameters: the electron mobility of NMOS channel $\mu =334\;{\mathrm{cm}}^{2}/\left(V\cdot s\right)$ (or the scattering time τ=49.4 fs), the parasitic source contact resistance $R_{s}=87\;\Omega$. These parameters are obtained by fitting the measured quasi-static drain-source resistance using the procedure described in [26]. The obtained electron mobility is in good agreement with the values that are commonly cited in the literature as typical ones for Si of comparable doping [27]. The used circuit model employs common simplified treatment of antenna electrical properties and uses the frequency-independent antenna parameters (such as directivity and efficiency) [21]. However, the actual antenna design has been accomplished using electromagnetic simulation tools and captured all EM aspects of the antenna and the substrate.

For the experimental determination of responsivity ${ℜ}_{V}$ and minimum optical noise equivalent power (NEP, $\mathrm{NEP}=V_{N}/{ℜ}_{V}$) of a TeraFET, we employed an electronic multiplier-chain source (more details on the measurement technique can be found in [26]). Our detector exhibits ${ℜ}_{V}∼100\;V/W$ optical voltage responsivity and NEP of 67 pW/$\sqrt{\mathrm{Hz}}$ at 0.6 THz. The responsivity was calculated as the ratio of detector radiation voltage response and the available power of the incoming signal. It should be pointed out that the presented values are the optical ones for the detector module, i.e., the total available beam power from the THz source (measured after all lenses, mirrors and the substrate) was used for the calculation. Hence, the optical losses introduced by the silicon lens and coupling of radiation to the integrated antenna are not deducted.

At low excitation intensities, the voltage response of a field effect transistor is proportional to the radiation power and can be generally expressed as:

(1) $$V_{\det}={ℜ}_{V}\cdot P_{\mathrm{THz}},$$

where $P_{\mathrm{THz}}$—the radiation power and ${ℜ}_{V}$ is the proportionality constant, usually called voltage responsivity and is measured in V/W. The constant ${ℜ}_{V}$ shall only depend on the detector characteristics but not on the power level, at least up to the point where saturation or higher-order effects begin to occur ([28], p. 213). In theory, the THz radiation detection with a FET is most often treated as the rectification of the modulated voltage applied between two terminals of FET. In small signal approximation, it results in a linear intensity dependence of the photoresponse [29,30,31,32], whereas a square root dependence is expected in a saturation at high radiation powers [33,34].

Recent experiments [6,35] have demonstrated that different FETs, such as Si MOSFETs (metal-oxide-semiconductor field-effect transistor) and AlGaN/GaN HEMTs (high-electron-mobility transistor) at the sub-threshold gate bias, when illuminated by a short-pulse of THz radiation, also exhibit a super-linear response slope before reaching saturation. In this regime, the voltage signal of the transistor is proportional to the radiation power as $V_{\det}\propto P_{\mathrm{THz}}^{n}$, where the index *n* is greater than 1. In [6], we concluded that a super-linear response is a universal property of rectification at low gate bias voltages and high radiation intensities, just before saturation sets in. A super-linear response to high THz intensities was recently predicted for the Schottky diode detectors as well [36].

**Figure 2:**
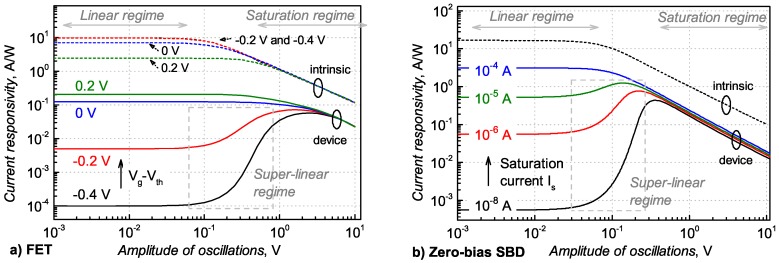
The simulated current responsivity for a field-effect-transistor (**a**) and a zero-bias Schottky barrier diode (**b**). Excitation power is proportional to the amplitude squared. Both devices exhibit three different regimes: a linear regime (the response is linearly proportional to the input power), a super-linear regime and a saturation regime. FET threshold voltage is 0.48 V and the diode non-ideality factor is 1.18.

In order to elucidate the underlying mechanism, we start from the unified charge carrier control model [37] which accounts for the nonlinear charge carrier concentration *n* dependency on the gate-to-source bias voltage $V_{G}$:

(2) $$n=\frac{C_{\mathrm{ox}}\eta V_{T}}{q}\cdot \ln\left[1+\frac{1}{2}\exp\left(\frac{V_{G}-V_{\mathrm{th}}}{\eta V_{T}}\right)\right],$$

where $V_{T}=k_{B}T/q$ is the thermal voltage with *q* being the elementary charge, $k_{B}$—Boltzmann constant, *T*—temperature, $V_{\mathrm{th}}$—the threshold voltage, $C_{\mathrm{ox}}$—the gate oxide capacitance per unit of area, η—the transistor ideality factor. Assuming that, in the biased transistor channel, the local carrier density depends on the gate-to-channel voltage difference $V_{G}-V_{\mathrm{ch}}\left(x\right)$ according to Equation (2), the dependency of the drain current $I_{d}$ on the gate and drain voltages obtain a simple analytic form:

(3) $$I_{d}=\frac{\eta^{2}V_{T}^{2}\mu C_{\mathrm{ox}}W}{L}\left[F_{1}\left(\frac{V_{G}-V_{\mathrm{th}}}{\eta V_{T}}-\ln2\right)-F_{1}\left(\frac{V_{G}-V_{\mathrm{th}}-V_{d}}{\eta V_{T}}-\ln2\right)\right],$$

where $F_{1}\left(x\right)=\int_{0}^{x}t/(\exp(t-x)+1)dt$ denotes the Fermi–Dirac integral, $V_{d}$—the drain voltage, *W* and *L*—respectively, the gate width and length, μ—the electron mobility. If we approximate the antenna as a voltage source with intrinsic resistance (for example 100 Ω), then, by applying harmonic voltage excitation, we can calculate the resulting rectified current. We can estimate responsivity in two ways: by normalizing the result by the power delivered to the antenna which will represent *a device responsivity* and the power absorbed by the transistor resulting in *an intrinsic responsivity*. The results of numerical calculations are presented in Figure 2 panels (a) for the transistor and (b) for an idealized diode which current–voltage characteristics is described by the Shockley equation: $I_{d}=I_{S}\left(\exp\left(V_{d}/\eta V_{T}\right)-1\right)$. For small signal excitation, the current responsivity is constant at all gate bias (FET case) or reverse-bias saturation currents $I_{S}$ (Schottky diode) values as expected for square-law power detectors. The rectification at large field amplitudes results in saturation of intrinsic responsivities (represented by dashed lines) for both devices. On the other hand, in the sub-threshold bias regime for FET and at low saturation currents for the Schottky diode, device responsivites when field amplitudes exceed about 100 mV, result in a super-linear dependency of responsivity. A direct comparison between the FET and Schottky diode shows that FETs posses a larger dynamical range where response is linear (up to two orders of applied power) and wider amplitude (and power) range where the device responsivity is super-linear.

## 3. Experiment Setup

**Figure 3:**
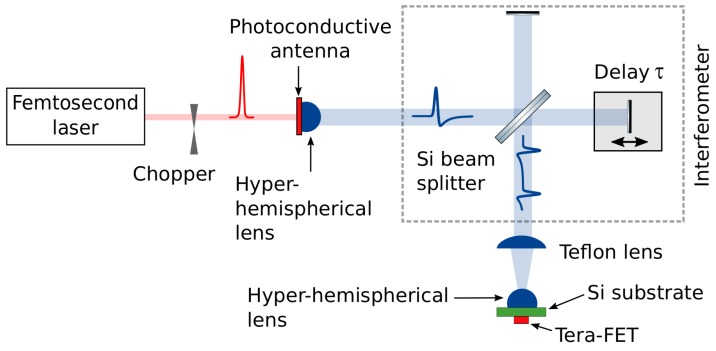
The THz autocorrelation setup. The Ti:sapphire oscillator generates 30 fs-long infrared pulses. The THz radiation power, emitted by a GaAs photoconductive emitter, is modulated through the rectangular-waveform bias. The response of THz detector is measured using a lock-in technique (not shown on diagram).

In order to test the validity of the TeraFET detector super-linear response phenomenon when excitated with ps-long THz pulses, we employ a photoconductive antenna as a THz radiation source driven by femtosecond-long Ti:sapphire laser pulses. In Figure 3, the setup is shown schematically. The laser emission was centered at λ = 800 nm and produced 30 fs-long pulses with a 75 MHz repetition rate. The ultrashort optical pulses were used to activate a free space photoconductive THz emitter from Teravil Ltd. (Vilnius, Lithuania) The GaAs photoconductive switch is fabricated by means of low temperature molecular beam epitaxy growth with characteristics of a few picoseconds carrier trapping time and about 2000 cm${}^{2}/V\cdot s$ electron mobility. On top of this epitaxial layer, AuGeNi coplanar microstrip contacts are formed with a 50 μm gap between them. These contacts were biased using a constant voltage source. The photosensitive THz emitter gap is illuminated with a train of femtosecond laser pulses that are tightly focused using a conventional lens. From the other side of the THz emitter chip, a hyper-hemispherical silicon lens was attached and positioned at the microstrip antenna center collimating the generated THz radiation and launching it into free space. The THz beam is modulated with a mechanical chopper enabling to employ the lock-in detection technique. The modulation frequency of 880 Hz was used for the autocorrelation measurements with CMOS FET and 20 Hz for the reference source power measurements with the Golay cell. The time constant of 300 ms was used in both cases.

**Figure 4:**
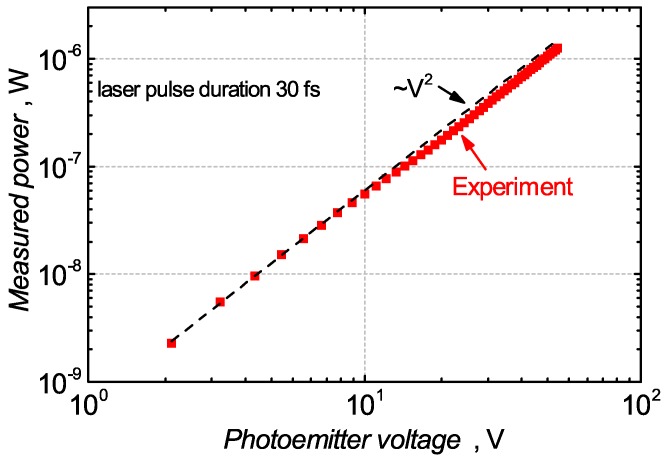
The average power of THz pulses measured with the Golay cell as a function of the photoconductive THz emitter bias voltage. The dashed line is the guide to an eye, a square law $P_{\mathrm{THz}}∼V^{2}$.

We started our investigations by measuring the generated THz radiation average power with the commercial Golay cell which was additionally calibrated using a photo-acoustic detector. Since the Golay cell is assumed to be a linear power detector at the measured frequencies, it lets us evaluate the THz source power dependence on the antenna bias voltage *V*. The measurement results are presented in Figure 4. The average power of terahertz radiation reached 1.25 μW at the antenna bias voltage of 50 V. The emitted THz power has a square-law proportionality on voltage bias, i.e., $P_{\mathrm{THz}}\propto V^{2}$.

## 4. Nonlinearity of the TeraFET Detector

**Figure 5:**
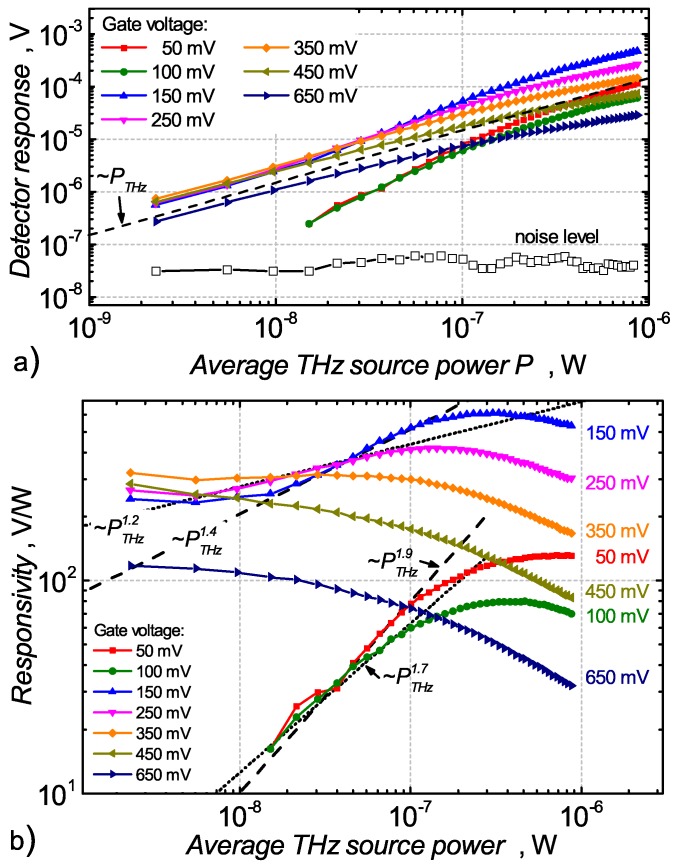
Rectified voltage (**a**) and responsivity (**b**) of the CMOS TeraFET detector exposed to a broadband pulsed THz radiation at various gate bias voltages. The threshold voltage of the FET is 480 mV. The noise data shown in panel (**a**) is a measurement result taken at 50 mV gate bias. The dashed lines are a guide to the eye—represent a law $V_{\det}∼P_{\mathrm{THz}}^{n}$.

The results of a TeraFET response on the excitation power level at different gate bias voltages are presented in Figure 5. The top panel shows a dynamic range of the measured responses, whereas Figure 5b presents a responsivity which equals the ratio between the response and the total available power. We have found that, for low averaged excitation powers above the gate threshold voltage $V_{\mathrm{th}}=445$ mV, the detected signal follows the antenna bias amplitude to the square law as expected from the linear dependence to power. The detector response gets into saturation at relatively low average power levels. Figure 5b shows that the widest range of linearity appears at $V_{G}$ = 350 mV which is slightly below the threshold voltage.

For the gate bias below 350 mV, we observe that the detected signal initially is linear to the power (corresponding to the small-signal regime) and then increases in a super-linear manner before entering into the saturation regime at higher excitation levels. At the gate bias of 150 mV, the response is approximately proportional to the exponent 1.4 of the beam intensity (or ∼$P_{\mathrm{THz}}^{1.4}$). The deeper into the sub-threshold regime, the steeper a power-vs-response curve becomes, i.e., the response is proportional to a higher exponent of the beam intensity. For the gate voltage of 50 mV, a nearly quadratic dependence (∼$P_{\mathrm{THz}}^{1.9}$) is observed.

Figure 5a also shows a detector noise level (black line with symbols) which allows for calculating the dynamic range of device sensitivity. For low excitation powers (less than 10 μW), the signal-to-noise ratio (SNR) is 10–15 dB. When THz radiation power increases, the SNR also grows and reaches 25–35 dB. We have implemented several noise suppression techniques such as shielding or removing ground loops in the setup, which lowered the detector noise level close to the theoretically expected thermal noise limit.

## 5. Nonlinear Autocorrelation Measurements

The basic principle of the autocorrelation measurement is the splitting of an incoming pulse into two arms and after introduction of a temporal shift between these copies superimposing them at the detector. For these purposes, we built a classical Michelson interferometer where a 400 μm thick high resistivity Si wafer was used as a beam-splitter. When the responsivity ${ℜ}_{V}$ does depend on power as given by Equation (1) and the detector response is instantaneous, the voltage signal of a FET can be defined by this formula [38,39]

(4) $$V_{\det}\left(\tau \right)\propto {ℜ}_{V}\int dt{\left(E\left(t\right)+E\left(t+\tau \right)\right)}^{2},$$

where E(t) and E(t+τ) is the signal and its time-delayed copy, τ—delay time. We can rearrange the equation as follows [4]:

(5) $$\begin{array}{cc} V_{\det}\left(\tau \right)\propto & {ℜ}_{V}\int dt\left(E{\left(t\right)}^{2}+E{\left(t+\tau \right)}^{2}\right)+\\ & +{ℜ}_{V}\int dt\left(E(t)E(t+\tau )+E(t)E(t+\tau )\right). \end{array}$$

The first term yields the total average power and comes as a constant background in the interferogram. The second term represents the interfering part of the autocorrelation signal and appears as peaks containing spectral information. The ratio of these two terms or the maximum of a real part and a background is called modulation depth, which in the case of linear autocorrelation is equal to 2:1. The integration in autocorrelation setups is typically realized with a lock-in amplifier possessing the time constant much longer than the pulse duration.

The autocorrelation performed with a linear detector allows for determining the frequency spectrum of THz pulse. However, the autocorrelation discards phase information, returning only the power, and does not therefore fit for determination of the intrinsic response speed of the detector itself as well as for the measurement of the THz pulse duration. The nonlinear detection process is needed. Then, the interferometric autocorrelation signal recorded by such a detector can be defined by this formula:

(6) $$V_{\det}\left(\tau \right)\propto \int dt{\left({\left(E\left(t\right)+E\left(t+\tau \right)\right)}^{2}\right)}^{n}.$$

**Figure 6:**
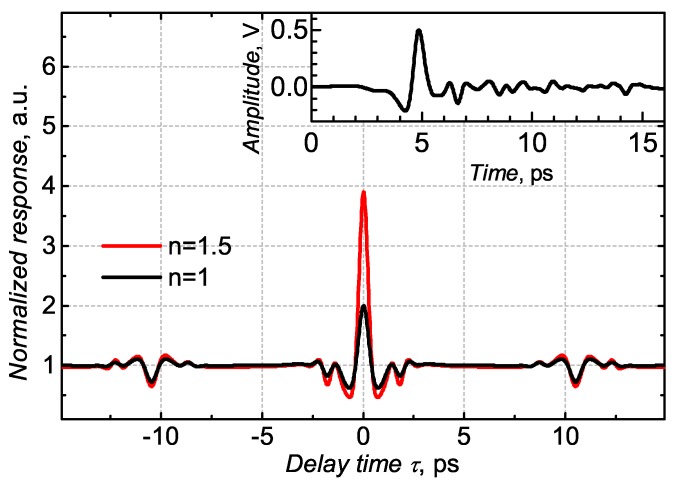
Modeled autocorrelation signal according to Equation (6) for two cases: a linear (*n* = 1) and a nonlinear (*n* = 1.5). The inset is the THz pulse measured with a photoconductive antenna.

Here, the index n>1. As we have already demonstrated, at the sub-threshold gate bias regime, the TeraFET can act as a super-linear THz detector. The deeper into the sub-threshold regime, the higher the number *n* that can reach two or even higher values. Consequently, the modulation depth is also larger than 2:1. A modeled example of such a nonlinear autocorrelation interferogram alongside with a linear signal is illustrated in Figure 6, assuming a temporal THz pulse shape as shown in the inset. The THz pulse was registered by a photoconductive antenna and deconvolved with the free carrier trapping time function of the epitaxial layer to obtain the actual THz pulse (more details in [40]). The etalon effect (see more below) is also included in the model.

**Figure 7:**
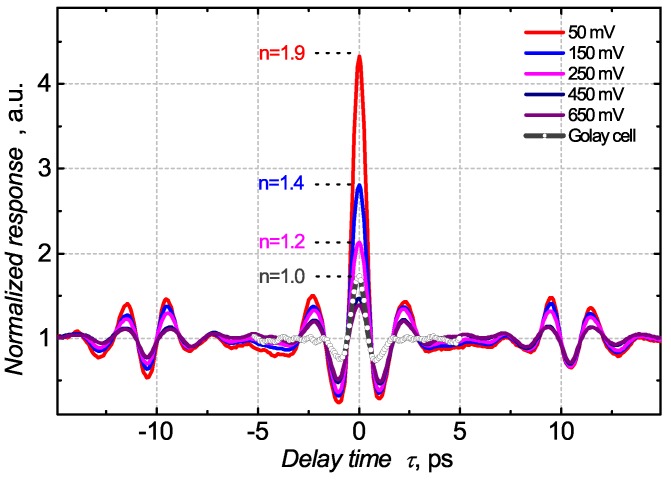
Measured autocorrelation traces of the CMOS TeraFET at different gate-voltages (solid lines) and the Golay cell (symbols) response. The sub-threshold bias regimes of the TeraFET are below 450 mV. The *n* numbers indicate the modeled exponent of relation between the detector response and the radiation power. The average power of a THz source is 0.87 μW.

Figure 7 presents autocorrelation interferograms for a TeraFET that are normalized to the non-interfering constant value. In a linear regime (above $V_{\mathrm{th}}=445$ mV), the normalized response does not depend on the gate voltage and overlaps in the diagram. The ratio between the signals measured at zero time delay and the temporally separated pulses is 1.5:1. A lower ratio (which is expected to be 2:1 in theory) could be explained by a non-ideal justification of an interferometer. The beam splitter function can also degrade the modulation depth. We also measured a reference interferogram with the Golay cell (dashed line in Figure 7). Due to its flat frequency response, this detector was expected to exhibit a near ideal, 2:1 modulation depth in the autocorrelation signal. However, our measurements revealed lower modulation depth of ≈1.7:1, indicating the necessity for further improvements of the spectrometer.

In the sub-threshold bias regime, the measured intensity autocorrelation trace exhibits a higher than 2:1 ratio due to the strong nonlinear response. The deeper into the sub-threshold regime, the greater the modulation depth observed, with the maximum being 4.5:1 at 50 mV gate voltage. The *n* numbers in Figure 7 indicate the exponent of the relation between the detector response and the radiation power. These data were obtained by fitting the curves in Figure 5 at the region of powers between 10 nW and 100 nW, where the super-linearity phenomenon manifests itself.

The autocorrelation traces are symmetric, since the pulse is convoluted with itself. However, the traces exhibit a sequence of additional peaks with a temporal delay of 12.5 ps. These peaks originate from the internal reflections in a 400 μm thick Si beam splitter named the etalon effect in the literature. Usually, this phenomenon is treated as a parasitic signal in time domain spectroscopy systems because it introduces false oscillations that interfere with data analysis and may obscure the important features of the spectrum [41,42]. One of the possibilities to avoid such reflections is by using an anti-reflection Cr coating for a Si beam splitter [41].

Our further efforts will be devoted to employment of the TeraFETs for spectroscopy applications [21]. On the other hand, the observed higher-order nonlinear phenomenon could be used as a convenient tool for monitoring of the temporal characteristics of THz pulses. In a pump-probe configuration, the nonlinearity of a TeraFET can also be used to determine the intrinsic response speed of the detector itself.

## 6. Conclusions

We have successfully employed an Si CMOS FET as a linear and nonlinear detector for the interferometric autocorrelation measurements driven by a photoconductive antenna as a THz radiation source. The maximum average power of the terahertz radiation was 1.25 μW behind an interferometer. We measured the response of CMOS FET detector versus the THz power of radiation and confirmed the existence of three gate voltage dependent working regimes of the TeraFET: a linear, a super-linear and a saturation. We have found that, in a wide range of gate bias voltages above the threshold voltage $V_{\mathrm{th}}=445$ mV, the detected signal follows the THz power linearly. This confirms that the TeraFET operates as a linear THz detector across a wide power range. For a gate bias below the threshold voltage (350 mV and less), the detected signal increases in a super-linear manner, as expected. All response regimes allow interferometric autocorrelation measurements; however, the modulation depth is by far the largest in the super-linear regime. We have shown that this mode is better suited for pulse characterization than the saturation. At a deep sub-threshold regime, the measured intensity autocorrelation trace exhibits a 4.5:1 ratio due to a strong nonlinear response. The nonlinearity of a TeraFET can be used to determine the intrinsic response speed of the detector itself as well as for the measurements of the THz pulse duration.
